# The Late Dr. D. C. O’Keefe

**Published:** 1871

**Authors:** 


### *The late Dr. D. C. O'Keefe.*

We are pained to chronicle the death of that distinguished physician, Dr. D. C. O'Keefe, who, after an illness of many weeks, died at Gainesville, Ga., on Tuesday, the 8th of August.

The action of the Academy of Medicine, of which he was a member, will appear in the next number, at which time we propose to give some events in a life that he made both honorable and useful.

At our request, we have been furnished with the following brief abstract of that portion of Rev. Dr. Brantly's address at the funeral service, which refers specially to the deceased:

Of him whom we are about to consign to the grave, it is not requisite that I should say much. I speak moreover to those who have known him well. He was a man of fine intellectual endowments, enriched by generous culture. He occupied a prominent place among the best physicians, both of our city and State. The large and lucrative practice he enjoyed among our most intelligent citizens, indicates the estimate in which he was held as a man of ability and discrimination. The lectures delivered to his class whilst Professor in the Atlanta Medical College were equal, in the judgment of the most intelligent alumni of the institution, to those of his most gifted contemporaries. He was a safe and vigorous thinker, and his thoughts were uniformly presented in a style both terse and perspicuous.

Dr. O'Keefe was a man of the kindest heart and quickest sympathies. His interest in his patients was intense; and few suffered more keenly in their sensibilities than he, when he found that his remedies were unavailing. A glance at his countenance would reveal at once his opinion of the patient. Joy or distress, written in his candid face, proclaimed the alternative of convalescence or deterioration. Such susceptibilities may not be desirable in a medical man—I allude to them as evincing the kind and tender heart of the *man*.

A day or two before he left the city for Gainesville, I met him in the office of a friend. "How are you, Doctor," I enquired, "how is it with you spiritually?" He replied, "I am not afraid to die—I am willing to die," or something of an equivalent import. "On what," I asked, "do you base this willingness? Is it because you are distressed in body, and anxious to be relieved from your sufferings, or is it because you have something better in expectation hereafter?" "The latter," was his reply. "Do you accept Jesus Christ as your Savior, and do you rely on his merits alone for salvation?" "That is my dependence. I was once an infidel, but the study of anatomy convinced me that there must be a God." This, so far as I now remember, was the substance of the last conversation I had with our friend. I thought that we might be speaking for the last time—as I then expected to leave the city before the time of his return—and I was anxious to deal candidly. But whatever we may say of him, whether in the accents of censure or praise, affects him not. He is beyond the reach of either. He had his faults; but I have chosen to speak of his virtues. His feeling nature attached many friends who were truly devoted, and who to-day mingle their sympathies with those who have suffered the deeper loss of one who, as husband and father, was all they could desire."

